# Prediction Model for Therapeutic Responses in Ovarian Cancer Patients using Paclitaxel-resistant Immune-related lncRNAs

**DOI:** 10.2174/0109298673281438231217151129

**Published:** 2024-02-14

**Authors:** Xin Li, Huiqiang Liu, Fanchen Wang, Jia Yuan, Wencai Guan, Guoxiong Xu

**Affiliations:** 1 Research Center for Clinical Medicine, Jinshan Hospital of Fudan University, Shanghai, 201508, China;; 2 Department of Oncology, Shanghai Medical College, Fudan University, Shanghai, 200032, China;; 3 Center for Tumor Diagnosis and Therapy, Jinshan Hospital, Fudan University, Shanghai, 201508, China

**Keywords:** Biomarker, chemoresistance, DEir-lncRNAs, immunotherapy, non-coding RNA, predicting tool, scRNA-seq

## Abstract

**Background:**

Ovarian cancer (OC) is the deadliest malignant tumor in women with a poor prognosis due to drug resistance and lack of prediction tools for therapeutic responses to anti-cancer drugs.

**Objective:**

The objective of this study was to launch a prediction model for therapeutic responses in OC patients.

**Methods:**

The RNA-seq technique was used to identify differentially expressed paclitaxel (PTX)-resistant lncRNAs (DE-lncRNAs). The Cancer Genome Atlas (TCGA)-OV and ImmPort database were used to obtain immune-related lncRNAs (ir-lncRNAs). Univariate, multivariate, and LASSO Cox regression analyses were performed to construct the prediction model. Kaplan-meier plotter, Principal Component Analysis (PCA), nomogram, immune function analysis, and therapeutic response were applied with Genomics of Drug Sensitivity in Cancer (GDSC), CIBERSORT, and TCGA databases. The biological functions were evaluated in the CCLE database and OC cells.

**Results:**

The RNA-seq defined 186 DE-lncRNAs between PTX-resistant A2780-PTX and PTX-sensitive A2780 cells. Through the analysis of the TCGA-OV database, 225 ir-lncRNAs were identified. Analyzing 186 DE-lncRNAs and 225 ir-lncRNAs using univariate, multivariate, and LASSO Cox regression analyses, 9 PTX-resistant immune-related lncRNAs (DEir-lncRNAs) acted as biomarkers were discovered as potential biomarkers in the prediction model. Single-cell RNA sequencing (scRNA-seq) data of OC confirmed the relevance of DEir-lncRNAs in immune responsiveness. Patients with a low prediction score had a promising prognosis, whereas patients with a high prediction score were more prone to evade immunotherapy and chemotherapy and had poor prognosis.

**Conclusion:**

The novel prediction model with 9 DEir-lncRNAs is a valuable tool for predicting immunotherapeutic and chemotherapeutic responses and prognosis of patients with OC.

## 1 INTRODUCTION

Ovarian cancer (OC) is the most lethal gynecological malignancy worldwide [1]. According to the American Cancer Society, there would be 19,710 new cases and 12,810 death cases of ovarian cancer in the United States [2]. Unfortunately, due to the lack of typical symptoms and early detective technology, most patients were diagnosed at a relatively late stage [3]. The common treatment for patients with OC is surgery combined with chemotherapy, such as cisplatin and paclitaxel (PTX) [1]. PTX has been used as a first-line chemo-drug since 1984 [4] and has been applied to advanced and refractory OC patients after primary surgery [5]. Furthermore, PTX alone is licensed to treat recurrent OC patients who cannot tolerate platinum-based drugs [6] and other cancers that are refractory to conventional chemotherapy [7]. However, patients with advanced OC often experience chemoresistance and recurrence, leading to an unfavorable prognosis [8]. The mechanisms underlying PTX resistance include the alteration of microtubules, drug transporters, cell cycle-related proteins, *etc*. [4]. Furthermore, immune therapies may not show satisfactory effects in patients with solid tumors [9]. Therefore, the discovery of reliable biomarkers for predicting chemotherapeutic and immunotherapeutic responses is of vital importance for patients with OC.

Long non-coding RNA (lncRNA), a category of transcripts longer than 200 nucleotides (nt), may have a significant impact on the responsiveness of PTX [10] and has been shown to be involved in metastasis, recurrence, and chemo-resistance of OC [11]. Emerging evidence revealed that lncRNAs, including N^6^-methyladenosine-related lncRNAs, ferroptosis-related lncRNAs, glycolysis-related lncRNAs, pyroptosis-related RNAs, and immune-related lncRNAs, can predict the prognosis of patients with OC [12-16]. However, most studies do not verify the prediction models through biological experiments.

The current study used RNA-seq analyses to identify differentially expressed PTX-resistant lncRNAs (DE-lncRNAs) between the PTX-resistant A2780-PTX cells and its parental PTX-sensitive A2780 OC cells. Combined with immune-related lncRNAs (ir-lncRNAs), PTX-resistant immune-related lncRNAs (DEir-lncRNAs) were discovered to construct a prediction model of therapeutic responses and prognosis in patients with OC and verify the efficacy based on the public drug response database and The Cancer Genome Atlas (TCGA) database.

## 2 MATERIALS AND METHODS

### 2.1 Cell Culture

All human-derived OC cells were examined for mycoplasma-free and authenticated using Short Tandem Repeat (STR) analysis. Ovarian endometrioid adenocarcinoma cell line A2780 and its counterpart PTX-resistant cell line A2780-PTX were obtained from Keygen Biotech (Nanjing, Jiangsu, China), and ovarian endometrioid adenocarcinoma cell line IGROV-1 was obtained from MilliporeSigma (Temecula, CA, USA). Cells were cultured in Dulbecco’s modified Eagle medium (DMEM, 4.5 g/L glucose) (Gibco, Invitrogen, Carlsbad, CA, USA) supplemented with 10% fetal bovine serum (FBS, Invitrogen).

### 2.2 RNA-Seq Analysis of Paclitaxel-resistant Ovarian Cancer Cells

Total RNA was extracted in A2780-PTX and A2780 OC cells. RNA-seq was performed and analyzed. The DE-lncRNAs between the above two cancer cell lines were sorted out by fold change (FC) >2 and *p<*0.001. To take into consideration outlier data, the raw data was processed by log10(count+0.001), followed by Z-score calculation, which was used for producing the heatmap.

### 2.3 Correlation Analysis of lncRNAs with Immune-related Genes

RNA transcriptome datasets and clinical information were downloaded from the Genomic Data Commons Data Portal (GDC Data Portal, https://portal.gdc.cancer.gov/) linked with the TCGA database. Genes were separated into protein-coding genes and lncRNA genes. DE-lncRNAs and ir-lncRNAs were obtained based on the expression matrix of lncRNAs in TCGA. The ir-lncRNAs were sorted by the analysis of the correlation between lncRNAs and immune-related genes using the Pearson Correlation Coefficient (|Pearson R|>0.5, and *p<*0.001) based on the ImmPort database (https://www.immport.org/home) and TCGA-OV datasets. A combination of DE-lncRNAs and ir-lncRNAs was used to construct a prediction model.

### 2.4 Construction of a Prediction Model

Data from TCGA-OV were classified into 2 sets: the training (control) group and the testing (validation) group. The differences between these two groups were then analyzed. After DE-lncRNAs and ir-lncRNAs were screened using univariate Cox regression analysis, followed by multivariate Cox regression analysis and LASSO Cox regression analysis, 9 prediction-model-related lncRNAs (DEir-lncRNAs) were obtained. The association of 9 DEir-lncRNAs with the overall survival (OS) of OC patients was examined using the “glmnet” R package and constructed the prediction model. The calculation of the prediction score (P-score) was as follows: P Score = (expression value of lncRNA#1 × coefficient of lncRNA#1) + (expression value of lncRNA#2 × coefficient of lncRNA#2) + … + (expression value of lncRNA#n × coefficient of lncRNA#n). According to a median P-score, patients were divided into high- and low-score groups.

### 2.5 Kaplan-Meier Survival Analysis and Principal Component Analysis (PCA)

The difference in OS rate between the high- and low-score groups was testified by Kaplan-Meier survival analysis in training (observing), testing (predicting), and total sets using the “survminer” R package. Principal component analysis (PCA) effectively visualized and separated identities from high- and low-score groups based on the expression data of all genes, DE-lncRNAs, ir-lncRNAs, and DEir-lncRNAs.

### 2.6 Independence and Validity of the Prediction Model

To exclude the influence of other clinical characteristics (age, grade, and stage), univariate and multivariate Cox regression analyses were used to verify the independent influence of the model on the OS rate of patients with OC. Concordance index (C-index) analysis was applied to compare the concordance of the prediction model with age, grade, and stage in predicting the OS rate of patients with OC using “rms” and “pec” R packages.

### 2.7 Nomogram

Clinical characteristics (age, stage, and grade) and P-scores were used to construct a nomogram for predicting the OS rate of patients with OC using the “rms” R package. The calibration curve verified the coherence between observed and predicted OS.

### 2.8 Prediction of Immune Function and Immunotherapeutic Responses

The enrichment of different immune functions and immune cells between high- and low-score groups was evaluated using “CIBERSORT”, “GSVA”, and “GSEABase” R packages. The scoring file for TCGA-OV was downloaded from the Tumor Immune Dysfunction and Exclusion (TIDE) database (http://tide.dfci.harvard.edu/). The immune-scoring data between high and low-score groups were analyzed using “limma” and “ggpubr” R packages. The correlation between immune fractions/immune cells and OS was analyzed using “survival” and “survminer” packages.

### 2.9 Prediction of Drug Sensitivity

The pRRophetic package was downloaded from GitHub (https://github.com/) and was used for the calculation of IC_50_ of various chemotherapeutic drugs between high- and low-score groups.

### 2.10 Co-expression Gene Analysis and Gene Set Enrichment Analysis (GSEA)

Functions between high- and low-score groups were analyzed using “enrichGO”, “GSVA”, and “GSEA” packages. Gene Ontology (GO) enrichment analyses, including biological processes (BP), cellular components (CC), and molecular function (MF) analyses were applied. GSEA was applied to analyze the top five pathways in high- and low-score groups.

### 2.11 EdU Assays

For the EdU (5-ethynyl-2’-deoxyuridine) assay, cells were seeded in a 24-well plate for 24 h, followed by labeling cells with EdU using the BeyoClickTM EdU-555 kit (Beyotime) according to the manufacturer's instructions. Cells were then photographed under the fluorescence microscope (OLYMPUS, Tokyo, Japan) at ×10 magnification. The ratio of red/blue was analyzed using Image J software (version 1.5.3a, National Institutes of Health, Bethesda, Maryland, USA) (http://imagej.nih.gov/ij).

### 2.12 Wound-healing, Migration, and Invasion Assays

A2780 and IGROV-1 cells were plated into 6-well plates and grew to 95% confluence. A sterile 200-μL pipette tip was used to scratch cells to make a wound. Images were captured at 0 and 96 h post-scratch, respectively, using the light microscope (OLYMPUS). For the migration assay, cells were directly plated into the upper chamber of a Transwell (Corning Inc. New York, NY, USA). For the invasion assay, the Transwell was coated with 5% Matrigel (BD, Franklin Lakes, NJ, USA) and incubated at 37°C for at least 8 h before cell plating. After cells were added to the upper chamber at the density of 8×10^4^ per well, the lower chamber was filled with medium containing 20% FBS, followed by incubation for 48 h. The cells on the upper side of the chamber were removed, whereas the cells on the bottom side of the chamber were then fixed and stained with Crystal violet solution (Sigma-Aldrich, Burlington, MA, USA) for 30 min, followed by photography at ×10 magnification using a light microscope (OLYMPUS). Finally, the number of cells was measured by ImageJ software.

### 2.13 Statistical Analysis

All data were analyzed by GraphPad Prism 8.0 (GraphPad Software Inc.) and presented as the mean ± SEM. The Student’s *t*-test was applied for a two-group comparison. The Wilcoxon rank-sum test and Spearman rank test were used to analyze the differences and correlations between the two groups, respectively. The Kaplan-Meier survival curve analysis was performed using the Log-rank test. The Cox hazard regression model was applied to calculate the hazard ratio (HR). Statistical significance was considered when *p<*0.05.

## 3 RESULTS

### 3.1 Characteristics of DEir-lncRNAs in Ovarian Cancer

**Fig. (1):**
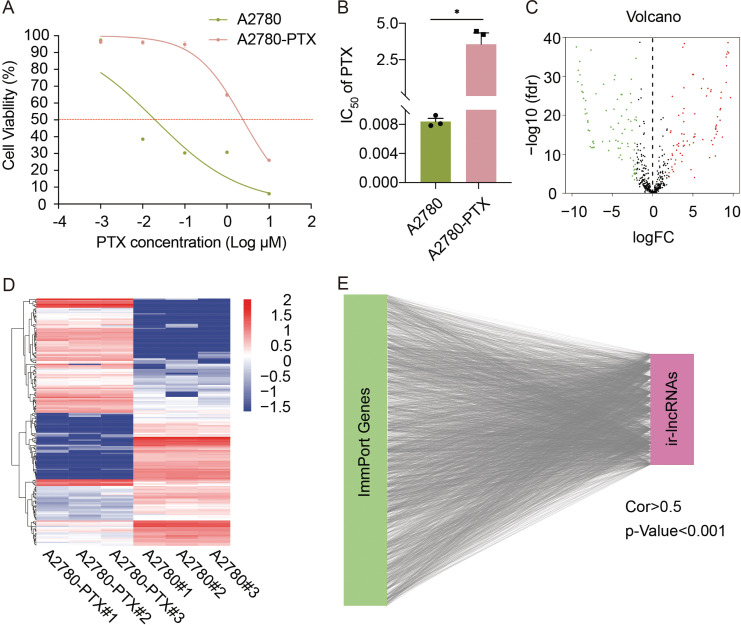
Analysis of paclitaxel-resistant associated and immune-related lncRNAs. (**A**) Measurement of cell viability in OC cells. The IC_50_ of PTX was detected in PTX-sensitive A2780 cells and their counterpart PTX-resistant A2780-PTX cells. (**B**) The IC_50_ of PTX in A2780 and A2780-PTX was calculated. The data are shown as the mean ± SEM; n = 3 independent experiments; Student’s *t*-test; *, *p <* 0.05. (**C**) Volcano plot of screened differentially expressed lncRNAs (DE-lcnRNAs) between A2780 and A2780-PTX cells (FC>2, *p<*0.001). (**D**) Heatmaps of the expression pattern of DE-lncRNAs in A2780 and A2780-PTX cells. (**E**) Identification of immune-related lncRNAs (ir-lncRNA). Pearson correlation analysis showed ir-lncRNAs correlated with immune-related genes from the ImmPort database (Cor>0.5, *p<*0.001) and plotted by Cytoscape. **Abbreviations:** FC, fold change; IC_50_, half-maximal inhibitory concentration; PTX, paclitaxel.

The whole workflow is shown in Fig. (**S1**). A2780 parental cells and PTX-resistant A2780-PTX cells were different in response to PTX evaluated by cell viability (Fig. **1A**) and measured by the IC_50_. The mean IC_50_ of PTX in A2780 and A2780-PTX was 0.0008±0.0004 and 3.565±0.7776 μM, respectively (Fig. **1B**). RNA-seq analysis showed that there were 186 DE-lncRNAs between A2780 and A278-PTX cells (FC>2 and *p<*0.001) identified from the TCGA-OV database (Fig. **1C**) and are listed in Table **S1**. The expression pattern of 186 DE-lncRNAs is shown in the Heatmap (Fig. **1D**). Pearson correlation analysis revealed that 225 ir-lncRNAs correlated with immune-related genes identified from the ImmPort database (|Pearson R|>0.5 and *p<*0.001) (Fig. **1E**). Next, the combination of DE-lncRNAs and ir-lncRNAs was used for the construction of a prediction model.

### 3.2 Construction of a Prediction Model and Verification of Prognostic Prediction in the TCGA-OV Database

**Fig. (2):**
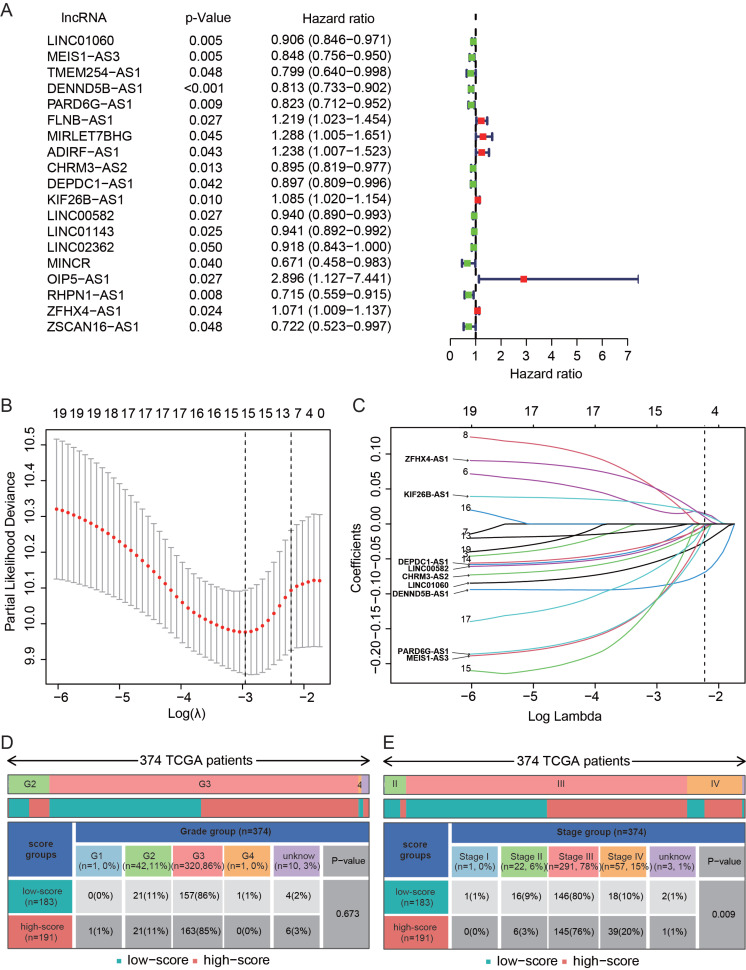
Construction of prediction model. (**A**) Forest plot of 19 prognosis-associated lncRNAs using univariate Cox analysis. Harzad ratio and 95% confidence interval are presented. (**B**) LASSO regression analysis for the construction of the prediction model. The partial likelihood deviance curve was plotted. The right dotted vertical lines were drawn at the optimal value by using the minimum criteria. (**C**) According to the optimal value of log(lambda), 9 lncRNAs in the prediction model were screened and shown. (**D**) Distribution of different grades of TCGA-OV dataset in high- and low-score groups. (**E**) Distribution of different stages of TCGA-OV dataset in high- and low-score groups. A *p*-value < 0.05 was considered statistically significant.

Samples from TCGA-OV were divided into the training (control, n=188) set and the testing (validation, n=186) set with equally distributed clinical features, including age, stages, and grades. No significant differences in clinical features between these two sets were observed except grades (*p* = 0.0134) (Table **S2**). Patients from the training set were used to construct a prediction model, whereas the testing set was used to verify the efficiency of the prediction model. First, using univariate Cox regression analysis of 186 DE-lncRNAs and 225 ir-lncRNAs, 19 lncRNAs associated with OS of OC patients were obtained (Fig. **2A**). Next, LASSO regression analysis was used for constructing the prediction model. These 19 lncRNAs were plotted by the partial likelihood deviance curve (Fig. **2B**), which was used consequently for the construction of the prediction model. Finally, to construct a prediction model, the LASSO Cox regression analysis was used and under the optimized selection, 9 DEir-lncRNAs (LINC01060, MEIS1-AS3, DENND5B-AS1, PARD6G-AS1, CHRM3-AS2, DEPDC1-AS1, KIF26B-AS1, LINC00582, and ZFHX4-AS1) were able to evaluate the P-score of patients from TCGA-OV (Fig. **2C**). The P-score of each patient was calculated based on the coefficients of the above 9 lncRNAs using a formula: P-score = LINC01060 × (-0.1031) + MEIS1-AS3 × (-0.2116) + DENND5B-AS1 × (-0.0976) + PARD6G-AS1 × (-0.1938) + CHRM3-AS2 × (-0.07511) + DEPDC1-AS1 × (-0.1155) + KIF26B-AS1 × (0.0508) + LINC00582 × (-0.0789) + ZFHX4-AS1 × (0.0958). Next, patients from the TCGA-OV dataset were divided into high- and low-score groups according to the P-score of each patient. Patients with different grades were almost equally distributed to high- and low-score groups (Fig. **2D**), and patients at stage IV accounted for 20% in the high-score group but 10% in the low-score group (Fig. **2E**), indicating that compared with the low-score group, patients in the high-score group had a higher probability in stage IV.

**Fig. (3):**
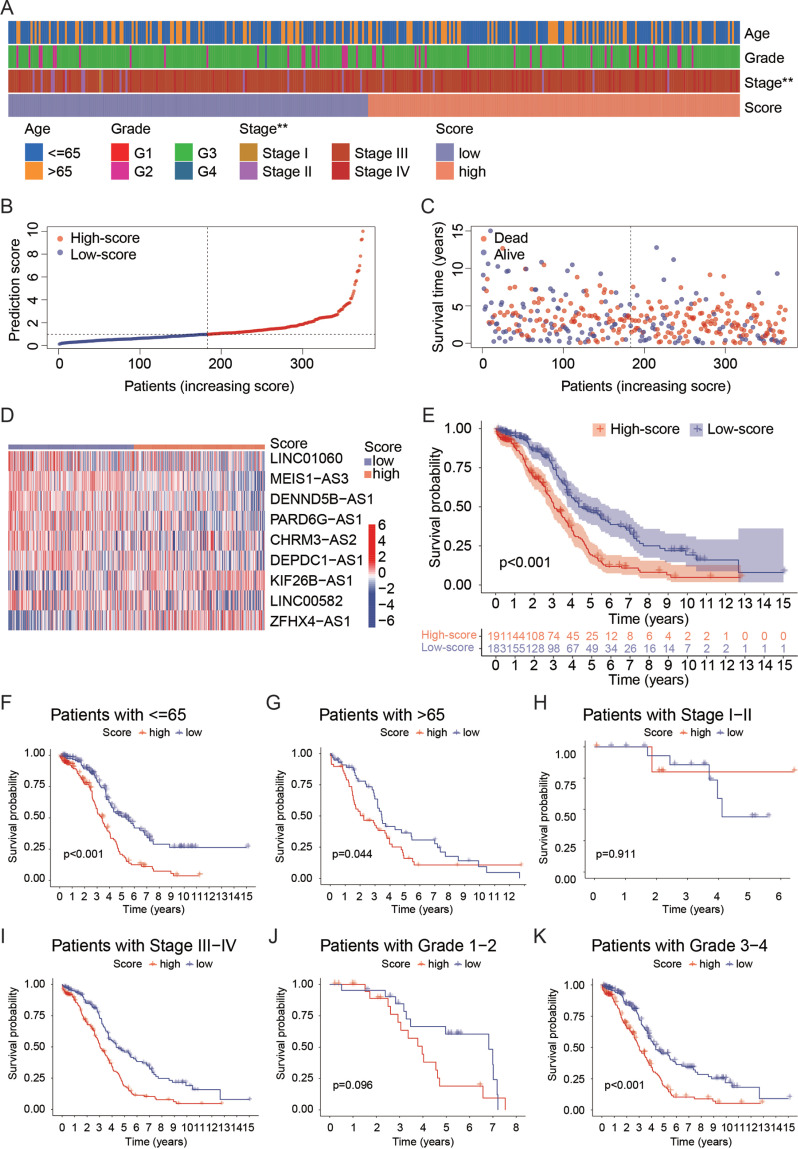
Correlation with clinical features and survival of the prediction model. (**A**) Heatmap showing distributions of age, grade, and stage subgroups of patients in high- and low-score groups. (**B**) Distribution of prediction scores between high- and low-score groups. (**C**) Distribution of survival time between high- and low-score groups. (**D**) Heatmap showing expression levels of prediction model-related lncRNAs in the whole TCGA-OV dataset. (**E**) Kaplan-Meier plot showing survival probability between high- and low-score groups. Kaplan-Meier plot showing survival probability in patients <=65 ages (**F**), >65 ages (**G**), stages I-II (**H**), stages II-IV (**I**), grades 1-2 (**J**), and grades 3-4 (**K**) between the high- and low-risk groups. A *p*-value < 0.05 was considered statistically significant.

An overall view of clinical features in high- and low-score groups is shown in the Heatmap (Fig. **3A**). The P-score and survival time of each patient are presented in Figs. (**3B**, **3C**), respectively. The pattern of each lncRNA expression between high and low-score groups is shown in the heatmap (Fig. **3D**). OS analysis showed that patients in a low-score group lived longer than those in a high-score group (Fig. **3E**). Survival time and status (Figs. **S2A**, **B**), P-score distribution (Figs. **S2C**, **D**), a heatmap of lncRNAs expression (Figs. **S2E**, **F**), and OS analysis (Figs. **S2G**, **H**) of the training and testing sets, respectively, further affirmed the efficiency of the prediction model in predicting the prognosis of patients with OC. Furthermore, a stratified analysis of age, grade, and stage was processed. The prognostic value of OS between high- and low-score groups was significantly different in patients regardless of age because both *p*-values in patients ≤65 and >65 ages were less than 0.05 (Fig. **3F**, **G**). The prognostic value of OS between high- and low-score groups was not different in patients with stages I-II (Fig. **3H**) but was significantly different in patients with stages III-IV (Fig. **3I**). The prognostic value of OS between high- and low-score groups was not different in patients with grades 1-2 (Fig. **3J**) but was significantly different in patients with grades 3-4 (Fig. **3K**). These data may suggest that the prediction model was able to use for predicting the survival of patients with OC in grades 3-4, and stages II-IV in all age groups. However, there was a limitation, such as the sample size of patients in low stages and grades being too small to have a reliable analytic result (Table **S2**).

### 3.3 Evaluation of the Association Between DEir-lncRNAs and Clinical Features of Patients in TCGA-OV

**Fig. (4):**
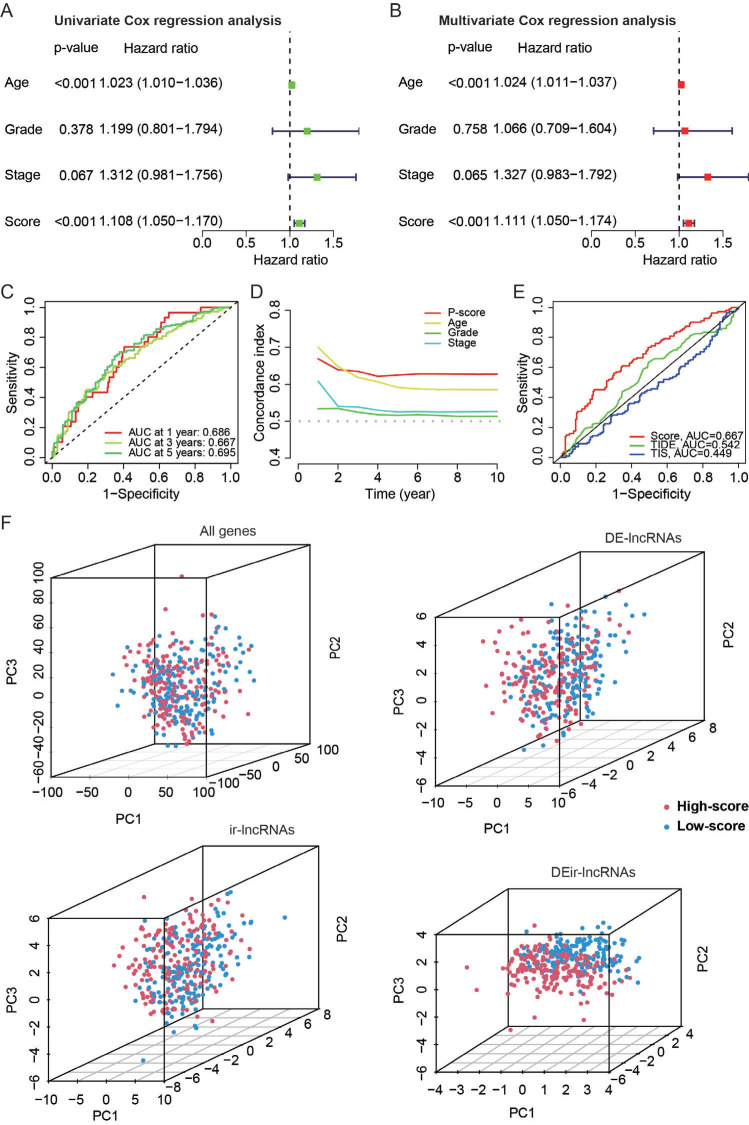
Validation of the prediction model and comparison with other predictive factors. Univariate Cox (**A**) and multivariate Cox analysis (**B**) of age, grade, stage, and risk scores in the TCGA-OV dataset. Harzad ratio and 95% confidence interval are presented. (**C**) Receiver Operating Characteristics (ROC) curve of the prediction model showing the Area Under The Curve (AUC) at 1, 3, and 5 years in predicting the prognosis of patients in the TCGA-OV dataset. (**D**) Concordance index (C-index) of the prediction model, age, grade, and stage. (**E**) ROC curve of the prediction model, Tumor Immune Dysfunction and Exclusion (TIDE), and Tumor Inflammation Signature (TIS) in predicting prognosis of patients in TCGA-OV dataset. (**F**) Component analysis of all genes, DE-lncRNAs, ir-lncRNAs, and DEir-lncRNAs in separating high- and low-score groups. PC, principal component. A *p*-value < 0.05 was considered statistically significant.

Whether DEir-lncRNAs were associated with the clinical features of patients was evaluated by Cox regression analyses. We found that prediction model-related lncRNAs were the independent prognostic biomarkers for patients with OC using univariate Cox regression (Fig. **4A**) and multivariate Cox regression analysis (Fig. **4B**). The hazard ratio (HR) for the P- score was 1.108 and 1.111 and the 95% confidence interval (CI) for the P-score was 1.050-1.170 and 1.050-1.174 by univariate Cox regression analysis (*p<*0.001) and multivariate Cox regression analysis (*p<*0.001), respectively. In addition, the receiver operator curve (ROC) analysis of the prediction model demonstrated that the area under the curve (AUC) at 1, 3, and 5 years was 0.686, 0.667, and 0.695, respectively (Fig. **4C**). These data indicate that the prognostic value of the prediction model was stable and reliable upto 5 years. The C-index of the P-score was consistently greater than that of other clinical features, such as age, grade, and stage in the prediction model (Fig. **4D**). Moreover, the AUC showed that our prediction model with the P-score was better than the prediction models of TIDE and Tumor Inflammation Signature (TIS) (Fig. **4E**). These data further suggest that the prediction model was a relatively reliable model for predicting prognosis using DEir-lncRNAs. Next, the ability to recognize the high-score identity of DEir-lncRNAs was verified by the principal component analysis (PCA). The PCA analyses revealed that all genes, DE-lncRNAs, and ir-lncRNAs had a relatively scattered distribution between high- and low-score groups, while DEir-lncRNA had a distinct distribution between high- and low-score groups (Fig. **4F**). These data indicate that the prediction model based on the above 9 DEir-lncRNAs can predict the prognosis of patients with OC.

### 3.4 Nomogram of Predicting Prognosis

**Fig. (5):**
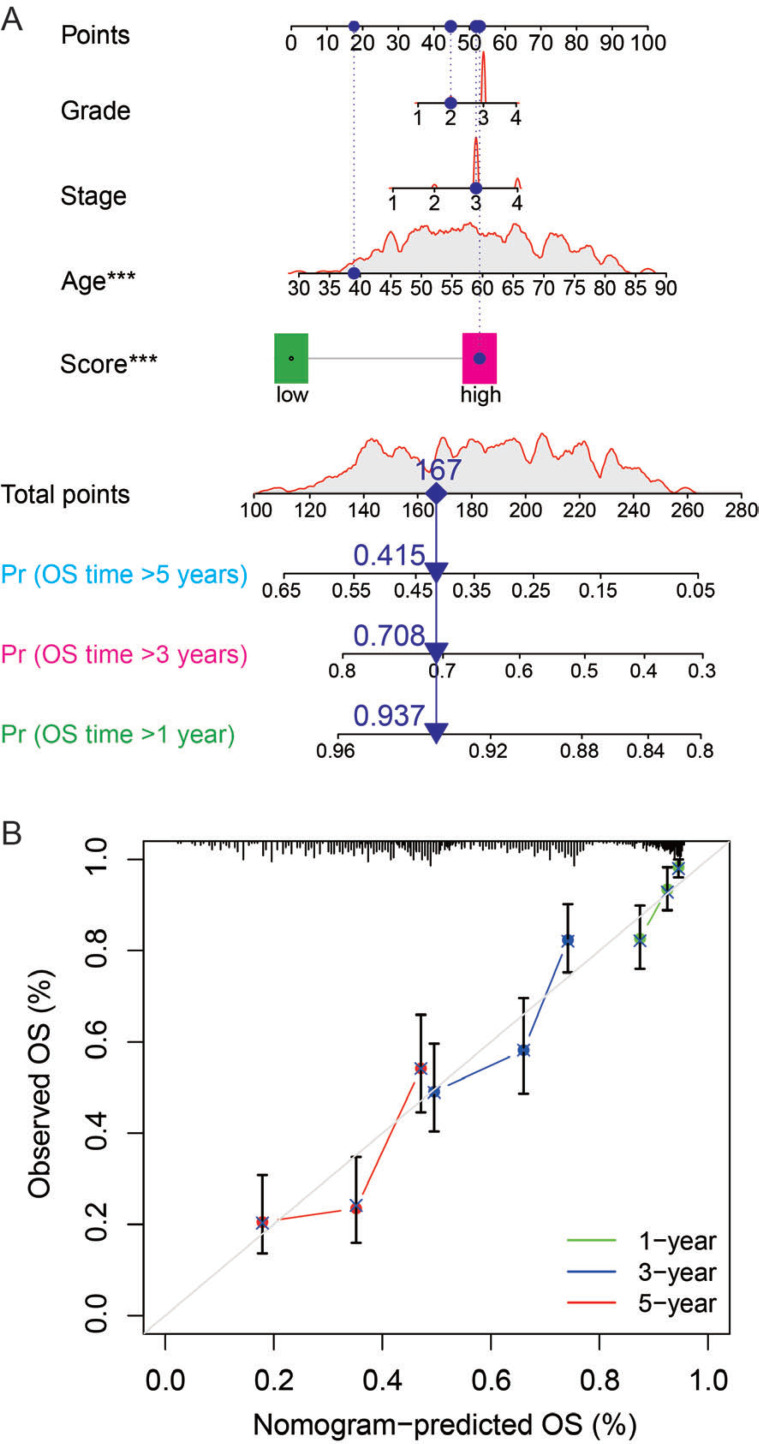
Nomogram of the prediction model. (**A**) The nomogram shows an example of how the points were calculated in a representative patient. The survival time over 1, 3, and 5 years was predicted using the age, grade, stage, and prediction scores of patients with ovarian cancer. The blue dots represent points of an OC patient with grade II, stage III, 40-year-old, and high-score status. The total point of the above factors is 167, and the probability of over 5-, 3-, and 1-year survival is 0.415, 0.708, and 0.937, respectively, for this patient. (**B**) Calibration for nomogram shows the predictions of 1-, 3-, and 5-year overall survival (OS) rates.

Constructing a nomogram with clinical characteristics and P-score was able to predict the 1-, 3-, and 5-year OS rate of patients with OC (Fig. **5A**), providing a potential application for clinical use. Calibration for nomogram showed that predictions for 1-, 3-, and 5-year OS rates were relatively precise with reference to the standard line (Fig. **5B**).

### 3.5 Comparison of Immune Functions between High- and Low-score Groups

**Fig. (6):**
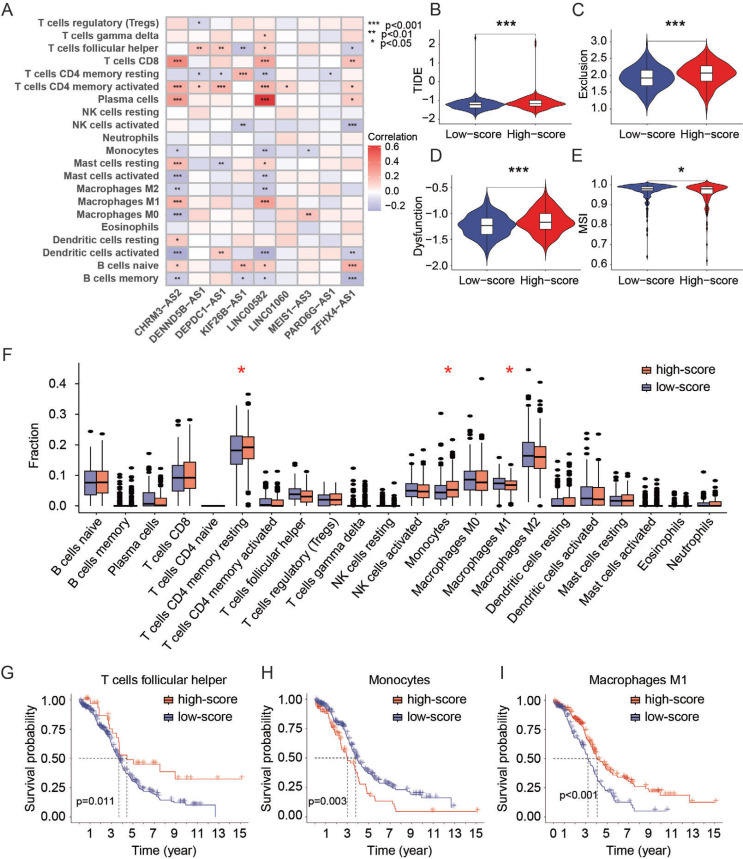
Immune correlations. (**A**) Correlation analysis between DEir-lncRNA and immune cells using the CIBERSORT database. Violin plot showing Tumor Immune Dysfunction and Exclusion (TIDE) (**B**), exclusion (**C**), dysfunction (**D**), and Microsatellite Instability (MSI) (**E**) scores between high- and low-score groups. (**F**) CIBERSORT analysis showing the comparison of immune fractions between high- and low-score groups. Kaplan-Meier plot showing the correlation of monocytes (**G**), T cells follicular helper (**H**), and macrophages (**I**) with the survival of patients between high- and low-score groups. *, *p <* 0.05; ***, *p <* 0.001.

To find potential applications of the prediction model with 9 DEir-lncRNAs, the immune functions between the high and low-score groups were analyzed. First, the correlation between each DEir-lncRNA and immune cells was analyzed based on the CIBERSORT database (Fig. **6A**). CHRM3-AS2 and LINC00582 were more relevant with immune cells, which may perform important roles in ovarian tumor immune microenvironment. Second, we utilized TIDE, an effective online database, to measure the rates of TIDE, T-cell exclusion, T-cell dysfunction, and microsatellite instability (MSI) of patients' data extracted from TCGA. We found that the TIDE rate was higher in a high-score group than in a low-score group (Fig. **6B**). Similarly, a higher exclusion rate (Fig. **6C**) and dysfunction rate (Fig. **6D**) were also observed in the high-score group compared to a low-score group. These data indicated that the high prediction score may be an indicator of the poor efficacy of immune checkpoint inhibitors (ICIs). Patients with OC in the high-score group would be more prone to evade immune therapy. Further analysis showed that the MSI rate was lower in the high-score group (Fig. **6E**), and a high MSI rate may indicate potential responsiveness to ICIs. Thus, 9 DEir-lncRNA could be predictive markers for responses to ICIs. The fraction of CD4 follicular helper T cells, monocytes, and macrophages M1 between high- and low-score groups was different in patients with OC based on the CIBERSORT database (Fig. **6F**). Furthermore, the difference in CD4 follicular helper T cells (Fig. **6G**), monocytes (Fig. **6H**), and macrophages M1 (Fig. **6I**) was related to the OS of patients with OC. Significant differences also existed in type II IFN (interferon) response and MHC class I (Fig. **S3A**) according to published 29-immune-signature genesets. The low-score group had a higher immune function rate in MHC class I, which was related to a better OS in patients with OC (Fig. **S3B**). Furthermore, to predict responsiveness to chemotherapeutic drugs, we performed the Wilcox test to measure the drug IC_50_ between high- and low-score groups using the “pRRophetic” R package. Consistently with the prediction model-based DE-lncRNAs, the IC_50_ of other chemo-drugs, such as cisplatin, methotrexate, doxorubicin, and vinorelbine, was higher in the high-score group than in the low-score group (Figs. **S4A**-**D**). Thus, we can predict the responses to the above chemotherapeutic drugs of OC using this prediction model of DEir-lncRNAs.

### 3.6 Confirmation of the Expression of DEir-lncRNAs in OC by scRNA-seq

**Fig. (7):**
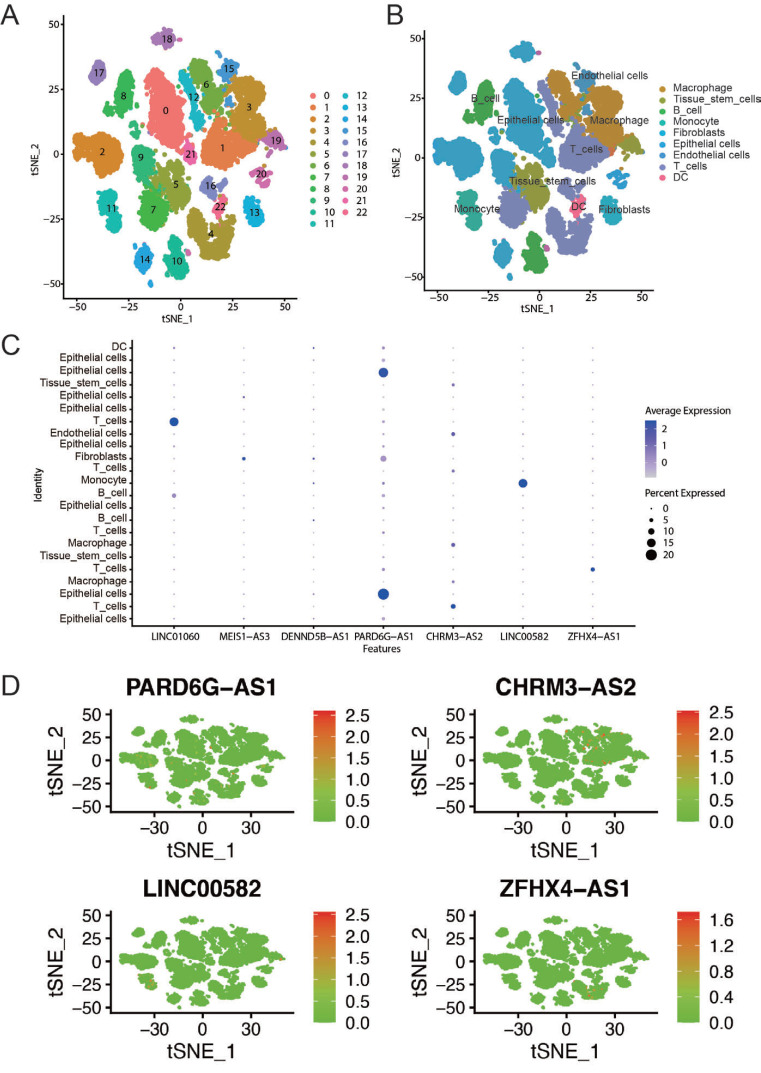
Expression patterns of DEir-lncRNAs in scRNA-seq data of OC. (**A**) tSNE plot of 23 cell clusters from GSE192898 scRNA-seq data. (**B**) Annotation of the 23 cell clusters. (**C**) Bubble plot showing the expression patterns of DEir-lncRNAs in different cell types. (**D**) Representative plot showing the distribution of different cell types of OC. t-SNE, t-distributed stochastic neighbor embedding.

Single-cell RNA sequencing (scRNA-seq) data of OC was obtained from GSE192898. A total of 18299 cells were used for the consequent data screening by the percentage of mitochondrial genes and features of intracellular genes, and 23 cell clusters were obtained (Fig. **7A**). According to the annotation tool ‘SingleR’, the specific cell type of each cell cluster was distinguished, including macrophage, tissue stem cells, B cell, monocyte, fibroblasts, epithelial cells, endothelial cells, T cells, and dendritic cell (DC) (Fig. **7B**). Bubble plot and t-SNE revealed that the expression patterns of DEir-lncRNAs were diverse in different cell types (Fig. **7C**, **D**). Among these, LINC01060, PARD6G-AS1, CHRM3-AS2, and ZFHX4-AS1 were highly expressed in T cells, whereas LINC01060 was also highly expressed in B cells and LINC00582 was enriched in monocytes.

### 3.7 Biological Function Enrichment and *In vitro* Validation

**Fig. (8):**
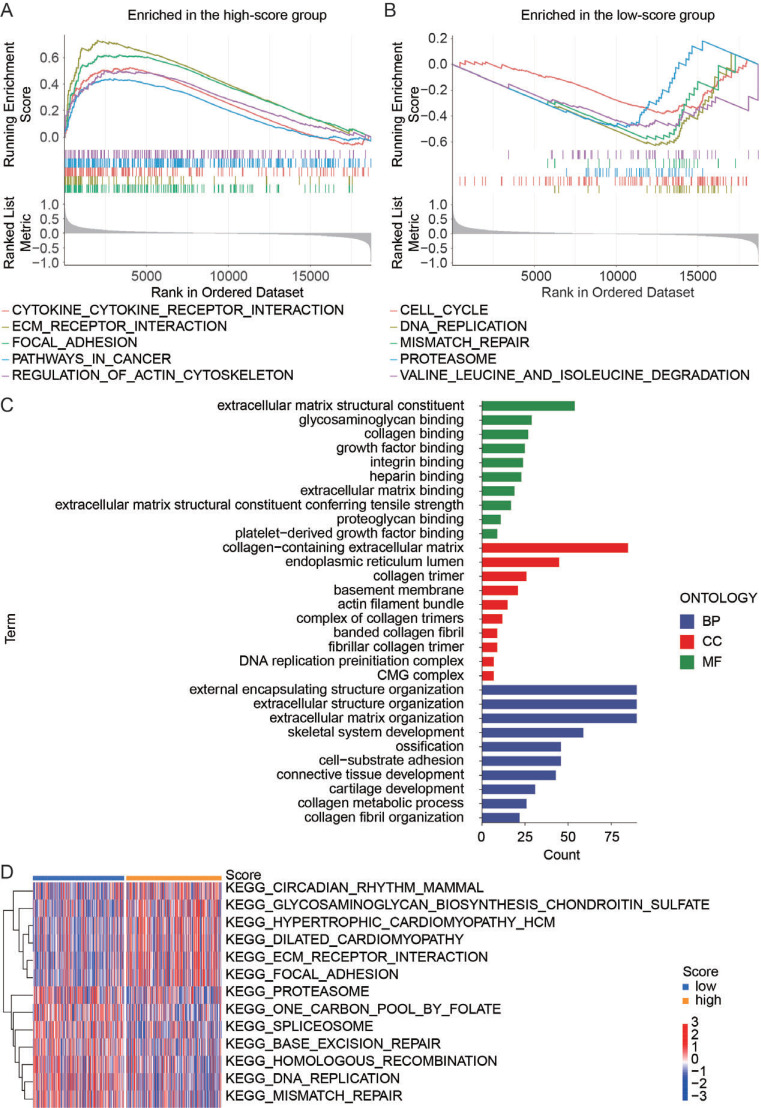
A gene set enrichment analysis (GSEA). (**A**) GSEA in the high-score group. (**B**) GSEA in the low-score group. (**C**) Gene ontology enrichment analysis based on differentially expressed genes between high- and low-score groups. (**D**) Gene Set Variation Analysis (GSVA) between high- and low-score groups. **Abbreviations: ** BP, biological process; CC, cellular component; MF, molecular function.

Besides the difference in prognostic value, immune function, and drug sensitivity between the high- and low-score groups, we analyzed GO and GSEA enrichment to find the biological distinction between these two groups. Cytokine receptor interaction, ECM receptor interaction, focal adhesion, *etc*., involved in metastasis were enriched in the high-score group (Fig. **8A**), whereas cell cycle, DNA replication, mismatch repair, *etc*. involved in tumor growth were enriched in the low-score group (Fig. **8B**). The GO term analyses showed that the enrichments of ECM structural constituent, collagen-containing extracellular matrix, external encapsulating structure organization, *etc*. were different between high- and low-score groups (Fig. **8C**), suggesting possible involvement in the metastasis and survival status. Gene Set Variation Analysis (GSVA) of the KEGG signature also showed differences between high and low-score groups in ECM receptor interaction, focal adhesion, DNA replication, mismatch repair, *etc*. (Fig. **8D**).

**Fig. (9):**
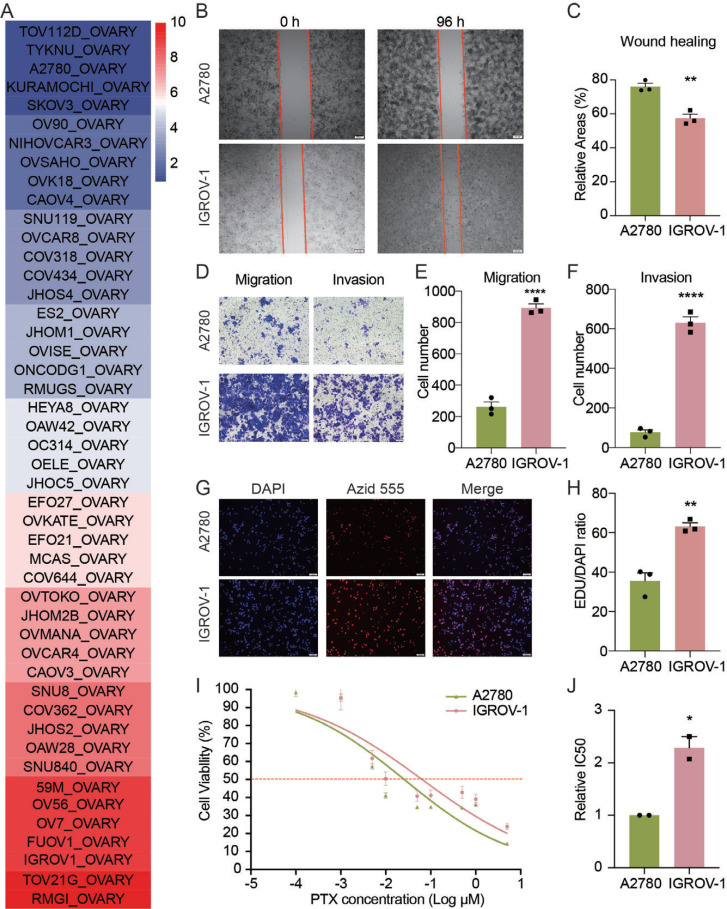
Validation in cell lines. (**A**) Heatmap of prediction scores of OC cell lines using the prediction model and Cancer Cell Line Encyclopedia database. (**B**, **C**) Experiments and statistical analysis of wound healing assay in A2780 and IGROV-1 cells. Scar bar, 200 μM. (**D**-**F**) Migration and invasion assay in A2780 and IGROV-1 cells. Scar bar, 100 μM. (**G**, **H**) Edu assay in A2780 and IGROV-1 cells. Scar bar, 100 μM. (**I**) Measurement of cell viability in A2780 and IGROV-1 cells. (**J**) Detection of IC_50_ of PTX in A2780 and IGROV-1 cells. The data are shown as the mean ± SEM. n = 3 independent experiments; Student’s *t*-test; *, *p <* 0.05; **, *p <* 0.01; ****, *p <*0.0001.

To verify the difference in biological functions between the two groups, we calculated the P-scores of ovarian cancer cell lines based on the Cancer Cell Line Encyclopedia (CCLE) database. The heatmap represented the P-scores of various OC cell lines (Fig. **9A**). Next, two common-used cell lines, A2780 (higher P-score cells) and IGROV1 (lower P-score cells), were chosen for validation and comparison of wound healing, migration, invasion, EdU, and IC_50_ of PTX. We found that IGROV-1 cells migrated faster than A2780 cells (Fig. **9B**, **C**). Consistently, the transwell assay demonstrated that IGROV-1 cells had a better ability in migration and invasion (Fig. **9D**-**F**). The EdU assay showed that DNA replication was more in the IGROV-1 cells than in the A2780 cells (Fig. **9G**, **H**), indicating a higher growth rate in IGROV-1 cells compared to A780. Furthermore, the IC_50_ of PTX was higher in IGROV-1 cells than in A2780 cells, suggesting that IGROV-1 cells with a higher P-score were more resistant to PTX than A2780 cells with a lower P-score (Fig. **9I**, **J**).

## 4 DISCUSSION

Patients with OC are often diagnosed at the late stage of the disease, and the advanced disease has a high mortality [17, 18]. Recurrences and resistance to chemotherapy severely influence therapeutic efficacy and lead to poor prognosis [19, 20]. The current study established a novel prediction model constructed using chemoresistant immune-related lncRNAs as biomarkers, which can be used for predicting the responsiveness of chemotherapy and immunotherapy as well as prognosis in patients with OC.

The standard treatment of OC is the combination of platinum and PTX after debulking surgery. PTX is a first-line chemo-drug because of its efficacy in primary treatment and low costs. However, some OC patients may face resistance to PTX and recurrence that severely influence the prognosis and threaten the life of patients. Previous studies have reported the involvement of lncRNAs in PTX resistance [21, 22] and the influence of immune-related lncRNAs in the prognosis [23, 24]. However, the role of PTX-resistant and immune-related lncRNA in OC remains unknown. Therefore, the discovery of biomarkers for predicting prognosis and resistance is important. Up to now, predictive studies for the prognosis have commonly focused on clinical features [25-27], serological parameters [28-31], imaging examination [32, 33], and combined biomarkers [29, 34]. Generally, serological parameters include typical CA-125, HE4, ctDNA, and gene-cluster signatures [35, 36]. In neoadjuvant chemotherapy, the normalization of CA-125 could predict the risk of recurrence [28]. FDA recommends that monitoring CA-125 could reflect chemotherapy responses and recurrence [37]. HE4 has been reported to correlate with responses to treatment [38]. ctDNA is used to predict the recurrence of OC after primary surgery [31]. Besides, recent studies focus more on gene-signatures prediction models, including nucleotide excision repair [36], endoplasmic reticulum stress [39], immune, pyroptosis [40], *etc.* (Tables **S3** and **S4**). The current study through the data analyses demonstrated that the 9 DEir-lncRNAs can be used as prediction biomarkers and we applied them to construct a novel prediction model for predicting prognosis, immune function, and therapeutic responses in patients with OC.

Based on our RNA-seq assay, 186 DE-lncRNAs were identified, and the expression matrix of these lncRNAs was then acknowledged in the TCGA-OV database. The correlation of immune-related genes and DE-lncRNAs was further analyzed by LASSO Cox regression analysis and 9 DEir-lncRNAs (LINC01060, MEIS1-AS3, DENND5B-AS1, PARD6G-AS1, CHRM3-AS2, DEPDC1-AS1, KIF26B-AS1, LINC00582, and ZFHX4-AS1) were applied to construct the prediction model. Among those DEir-lncRNAs, ZFHX4-AS1 was reported to be upregulated in OC and is associated with tumor-infiltrating immune cells [41]. A decrease in ZFHX4-AS1 expression may be favorable to the prognosis of patients with OC. Decreased expression of LINC01060 has been reported to be associated with the progression of pancreatic cancer by vinculin-mediated focal adhesion turnover [42]. LINC01060-containing exosomes can promote the progression of glioma [43]. In addition, LINC01060 is predicted as CD4^+^ T cell-related lncRNA and is involved in the prediction of the prognosis of hepatocellular carcinoma [44]. However, the exact correlation between LINC01060 and the prognosis of patients with OC has not been identified. KIF26B-AS1 promoting the malignant progression of laryngeal cancer was also reported [45]. The current study showed for the first time that MEIS1-AS3 is proven in predicting prognosis in OC but the role of MEIS1-AS3 on chemoresistance in OC remains to be explored. Single-cell RNA sequencing (scRNA-seq) data of OC confirmed the expression and the relevance of DEir-lncRNAs in immune cells and responsiveness.

The prediction scores of each patient were calculated based on the expression level and relevant coefficients. For better comparison, we used the median prediction score to separate patients into high- and low-score groups in the prediction model. Following Kaplan-Meier analysis to validate the efficiency of 9 DEir-lncRNAs in predicting the OS rate of patients with OC in the training set, testing set, and entire samples, we demonstrated that patients in the low-score group had a more promising prognosis. The capability of the prediction model in patients was further testified by stratified OS rate analyses of age, grade, and stage. We found that the OS rate of patients of all ages, stages III-IV, and grades 3-4 was significantly different between high- and low-score groups. As the sample size was relatively small, the difference in the OS rate of patients with grades 1-2 and stages I-II between high- and low-score groups was not observed. In the future, increasing the sample size may overcome this limitation. Additionally, PCA analysis proved the remarkable ability of the prediction model to distinguish identities from high- and low-score groups. To exclude the influence of age, stage, and grade, univariate and multivariate Cox regression analyses were applied and demonstrated that 9 DEir-lncRNAs were independent prognostic biomarkers for patients with OC. The C-index and nomogram further verified the accuracy and precision of the OS rate in the prediction model. Indeed, our study exhibited that the evaluation for 1-, 3-, and 5-year OS rates were relatively consistent, and the calibration curve showed satisfied coherence between predicted and observed OS rates.

It has been shown that OC has modest responses to immunotherapy, while the combination of immunotherapy with typical chemotherapy has a significant impact on the efficiency of treatment [46]. Several checkpoint inhibitors are undergoing clinical trials in ovarian cancer, along with a combination of chemotherapy drugs [47, 48]. There is a strong association between IFN-γ and immune checkpoint blockade (ICB) responses [49]. Our prediction model identified type II IFN responses differently between high- and low-score groups, indicating that this model can be used to predict the feature of immune function. The analysis of TIDE, exclusion, and dysfunction further proved that patients in the low-score group had a better response to immunotherapy, and DEir-lncRNA could be predictive markers for responses to ICIs, similar to immune checkpoint markers [50]. It has been reported that mismatch repair deficient cancers are more sensitive to ICIs, like anti-PD-1/PD-L1 drugs [51]. Our data suggest that patients in the high-score group may be more prone to evade immunotherapy.

OC patients are often faced with resistance and recurrence, causing poor prognosis. Although our prediction model is initially constructed for predicting PTX resistance, indeed, this model can also be used for predicting other chemotherapeutic drugs, such as cisplatin, methotrexate, vinorelbine, and doxorubicin, which have previously been reported to be effective in clinical application. Consistently with results from the prediction model, the IC_50_ of cisplatin, methotrexate, vinorelbine, and doxorubicin was higher in the high-score group than in the low-score group. It has been shown that lncRNAs can be induced by cisplatin as well as PTX in OC [52, 53]. The current study also observed a higher P-score in patients who were resistant to methotrexate. Methotrexate has been used to treat certain types of cancer, such as folate receptor (FR)- positive OC patients [54] and lncRNAs are involved in methotrexate resistance [55]. Our prediction mode also showed that patients with a higher P-score were more resistant to doxorubicin and vinorelbine compared to patients with a low P-score. Doxorubicin has been proven for systemic therapy in OC patients [56], and dysregulated lncRNAs are not beneficial from treatment [57]. Vinorelbine has been used for relapsed OC [58, 59]. The resistance of vinorelbine has been found in breast cancer, lung cancer, and mesothelioma patients [60-62], but the involvement of lncRNAs in their resistance has not been explored yet. Our data consequently indicate the significance of the prediction model in chemotherapeutic and immunotherapeutic responses and prognosis of OC patients. Targeting these molecular abnormalities would bring us closer to the goal of personalized therapy and may improve the prognosis for patients with OC [63].

The current study has some limitations. For instance, the sample size of patients in low stages and grades is too small. Although the prediction model constructed by nine-DEirlncRNAs shows predictive capacity and has been confirmed in OC cell lines, it indeed lacks verification by large-scale clinical OC cohort. Currently, immune checkpoint inhibitors show limited responses for OC; therefore, RNA-seq data from the immune therapy cohort alone are inadequate for testifying the prediction model. For further directions, the collection of blood samples and tumor tissues from OC patients can be utilized to testify to the real-world feasibility. Consequently, a clinical trial is needed after being consented to by the Ethics Committee when patients are faced with chemoresistance and recurrence.

## CONCLUSION

Based on the RNA-seq analysis, the differentially expressed PTX-resistant lncRNAs between A2780-PTX and A2780 cells were identified. Combined with immune-related lncRNAs, the DEir-lncRNA-related prediction model was generated. The novel prediction model with 9 DEir-lncRNAs is a valuable tool for predicting chemotherapeutic and immunotherapeutic responses and prognosis in patients with OC and may provide a potential application for clinical use.

## AUTHORS’ CONTRIBUTIONS

X.L. and G.X. conceived the study. X.L., H.L., F.W., J.Y. and W.G. performed experiments, bioinformatics analyses, and validation. G.X. acquired funding. X.L. wrote the original draft. G.X. administrated the project and wrote, reviewed, and edited the manuscript. All authors reviewed the manuscript.

## Data Availability

The datasets supporting the findings of this study are included within the article and its Supplimentry material. More detailed data are available from the corresponding author upon reasonable request.

## LIST OF ABBREVIATIONS

DE-lncRNAs: Differentially Expressed Paclitaxel-resistant lncRNAs
DEir-lncRNAs: PTX-resistant Immune-related lncRNAs
IC_50_: Half-maximal Inhibitory Concentration
ICIs: Immune Checkpoint Inhibitors
ir-lncRNAs: Immune-related lncRNAs
OC: Ovarian Cancer
PTX: Paclitaxel
scRNA-seq: Single-cell RNA Sequencing
TIDE: Tumor Immune Dysfunction and Exclusion
TME: Tumor Microenvironment
