# Supplementary material for: Prediction Model for Therapeutic Responses in Ovarian Cancer Patients using Paclitaxel-resistant Immune-related lncRNAs
Source: Curr Med Chem. 2024 Feb 14;31(26):4213–31. doi: 10.2174/0109298673281438231217151129 (PMC11340295; doi:10.2174/0109298673281438231217151129)
Supplement: Supplementary file 1 [file CMC-31-4213_SD1.pdf]

Supplementary Material

Prediction Model for Therapeutic Responses in Ovarian Cancer Patients Using Paclitaxel-resistant Immune-related lncRNAs

Xin Li[1,2], Huiqiang Liu[1,2], Fanchen Wang[1,2], Jia Yuan[1,2], Wencai Guan[1] and Guoxiong Xu[1,2,3,*]

[1]Research Center for Clinical Medicine, Jinshan Hospital of Fudan University, Shanghai, 201508, China; [2]Department of Oncology, Shanghai Medical College, Fudan University, Shanghai, 200032, China; [3]Center for Tumor Diagnosis and Therapy, Jinshan Hospital, Fudan University, Shanghai, 201508, China

Additional file 1-Table S1

geneNames	**logFC**	**logCPM**	**LR**	**PValue**	**FDR**
XIST	-14.793339	8.40282922	1401.544	9.70E-307	6.91E-305
DNM3OS	-13.818311	5.5350056	1478.594	1.98E-323	1.68E-321
TRHDE-AS1	-12.231658	3.95667045	878.8645	3.86E-193	1.12E-191
FLJ36000	-12.023888	3.7509208	776.5713	6.70E-171	1.64E-169
TBX2-AS1	-11.674538	3.40549914	688.7131	8.51E-152	1.82E-150
LINC00839	-11.597442	3.32872222	652.4428	6.57E-144	1.32E-142
MIR137HG	-11.270714	3.00956736	492.4538	4.17E-109	6.18E-108
LINC01597	-10.734901	2.48185283	394.5772	8.34E-88	1.01E-86
PCAT14	-10.667538	4.2873668	904.8645	8.60E-199	2.57E-197
FAM230C	-10.125891	1.89072386	281.6607	3.26E-63	2.97E-62
C8orf31	-10.073437	1.84184737	251.401	1.29E-56	1.06E-55
BISPR	-10.016251	1.78411055	259.9704	1.74E-58	1.48E-57
LINC00501	-9.9029244	1.67194937	244.4446	4.22E-55	3.43E-54
ZNF667-AS1	-9.8052383	1.57447252	224.0831	1.16E-50	8.84E-50
TSIX	-9.7986475	3.42022499	254.5908	2.59E-57	2.17E-56
LINC01060	-9.7492663	1.52385017	228.386	1.34E-51	1.04E-50
CCDC144NL-AS1	-9.7459685	4.15924626	1082.372	2.24E-237	8.67E-236
SALRNA1	-9.580016	1.36007158	195.6917	1.82E-44	1.26E-43
LINC01139	-9.4470672	1.23667588	170.9883	4.50E-39	2.85E-38
RFPL1S	-9.2768434	1.0748137	129.7943	4.55E-30	2.37E-29
NR2F1-AS1	-9.2293859	2.86698081	501.3194	4.91E-111	7.41E-110
LINC01224	-9.2211874	1.02188254	143.2492	5.19E-33	2.90E-32
TRG-AS1	-9.0315231	0.84316147	153.8211	2.53E-35	1.49E-34
LINC01097	-9.0151824	0.83606754	137.9364	7.52E-32	4.09E-31
TP53TG1	-8.767199	0.59836668	126.1135	2.90E-29	1.49E-28
STARD4-AS1	-8.7609187	0.58683026	118.321	1.47E-27	7.25E-27

AF131215.2	-8.6435512	0.47240716	117.02	2.84E-27	1.39E-26
LINC00210	-8.5641882	0.41060304	109.9694	9.95E-26	4.65E-25
SATB1-AS1	-8.356065	0.22338837	97.17988	6.33E-23	2.74E-22
KIF25-AS1	-8.3518053	0.2222581	91.60145	1.06E-21	4.43E-21
LINC00623	-8.1637498	1.82927184	279.576	9.29E-63	8.40E-62
PCED1B-AS1	-8.1539413	0.03676158	92.37353	7.18E-22	3.01E-21
LINC01515	-8.0366631	-0.0721048	83.55813	6.19E-20	2.41E-19
LINC01391	-8.0239587	-0.0847152	81.76824	1.53E-19	5.88E-19
MEIS1-AS3	-7.8200922	-0.2639791	71.59336	2.64E-17	9.40E-17
LINC01091	-7.6826414	2.13232122	300.4644	2.61E-67	2.50E-66
VIM-AS1	-7.6484997	2.59851456	167.2677	2.92E-38	1.83E-37
SNHG14	-7.5868594	5.36538351	1244.512	1.29E-272	6.46E-271
MIR7-3HG	-7.4257425	-0.6090178	58.31226	2.24E-14	7.00E-14
LINC01425	-7.3897033	-0.6310359	51.74549	6.32E-13	1.85E-12
AC012123.1	-7.3044855	-0.7069975	56.50632	5.60E-14	1.72E-13
AC079117.1	-7.2506773	-0.7487959	52.15785	5.12E-13	1.51E-12
WWC2-AS2	-7.0250747	0.75020436	115.4413	6.30E-27	3.04E-26
DLX6-AS1	-6.6675739	6.90129634	1390.314	2.67E-304	1.85E-302
LINC01138	-6.0885278	0.62285083	126.7186	2.14E-29	1.10E-28
FAM66C	-5.4571922	-0.6478867	48.17289	3.90E-12	1.10E-11
PCOLCE-AS1	-5.1695244	0.27064102	76.02594	2.80E-18	1.03E-17
AL589743.1	-4.8455934	3.77568609	559.0528	1.35E-123	2.28E-122
CYP4F26P	-4.7947607	0.5474965	97.15984	6.40E-23	2.77E-22
DUBR	-4.7442488	1.09926601	132.1164	1.41E-30	7.45E-30
TSPEAR-AS1	-4.6625158	1.31546214	137.2667	1.05E-31	5.71E-31
TUBA3FP	-4.5591206	0.57165952	60.29304	8.17E-15	2.61E-14
LINC01410	-4.3640369	1.05211347	108.8223	1.78E-25	8.26E-25
MIR17HG	-4.2876107	2.47231859	193.3716	5.84E-44	4.01E-43
VLDLR-AS1	-4.2849253	0.10422169	59.04413	1.54E-14	4.86E-14
DBH-AS1	-4.26898	-0.1807739	50.78925	1.03E-12	2.99E-12
DUXAP8	-4.2489423	0.28557557	80.49863	2.91E-19	1.11E-18
TMEM254-AS1	-4.1567984	-0.608901	39.93478	2.63E-10	6.83E-10
USP30-AS1	-4.0288788	0.0807899	50.82801	1.01E-12	2.93E-12
PEG13	-3.984155	-0.4212831	42.78464	6.11E-11	1.64E-10
HOXC-AS2	-3.9630253	3.30211056	335.6329	5.70E-75	5.97E-74
BOLA3-AS1	-3.8937535	2.31089645	114.7212	9.06E-27	4.35E-26
HOXC-AS1	-3.8464895	0.70552078	89.16448	3.63E-21	1.48E-20
DNAH10OS	-3.8080867	-0.3318909	37.51322	9.08E-10	2.30E-09
RDH10-AS1	-3.8070501	1.40992192	114.3936	1.07E-26	5.12E-26

FAM66B	-3.7934492	-0.1002651	56.58939	5.37E-14	1.65E-13
DENND5B-AS1	-3.7805011	0.76658981	68.55705	1.23E-16	4.27E-16
AP006222.2	-3.5050055	0.29642045	60.24298	8.38E-15	2.68E-14
LINC01341	-3.4532685	0.94491297	73.36559	1.08E-17	3.89E-17
AC006538.1	-3.1988348	1.18436892	94.41935	2.55E-22	1.09E-21
DLGAP1-AS1	-3.0900171	0.55769033	56.36207	6.03E-14	1.85E-13
PARD6G-AS1	-3.0400232	0.51689026	52.84936	3.60E-13	1.07E-12
CYP1B1-AS1	-3.0006063	1.68991058	84.82983	3.25E-20	1.28E-19
NBR2	-2.9347905	2.17677326	144.2785	3.09E-33	1.74E-32
AC159540.1	-2.7506495	1.81111743	115.5255	6.04E-27	2.92E-26
PCAT6	-2.7495972	0.10163361	39.36915	3.51E-10	9.06E-10
A1BG-AS1	-2.7072103	1.28471358	82.70938	9.50E-20	3.69E-19
LINC00662	-2.6408693	2.83537829	132.6686	1.07E-30	5.66E-30
IPO9-AS1	-2.5073319	0.09133512	18.19156	2.00E-05	3.86E-05
SGMS1-AS1	-2.3783368	0.97622941	36.35931	1.64E-09	4.10E-09
NRSN2-AS1	-2.3574138	0.36788742	29.30039	6.20E-08	1.42E-07
C9orf139	-2.3051306	-0.2458811	20.70796	5.35E-06	1.08E-05
AC060834.2	-2.2965679	-0.1514358	25.14325	5.32E-07	1.15E-06
PCAT7	-2.2670073	0.92856953	48.98472	2.58E-12	7.35E-12
MORC2-AS1	-2.2428198	0.27736123	13.86773	0.00019614	0.0003493
LINC00304	-2.2043633	1.41128869	56.7397	4.97E-14	1.53E-13
LINC00265	-2.2032012	2.06835261	84.87744	3.17E-20	1.25E-19
KCNQ1OT1	-2.1910782	3.49821765	184.4975	5.05E-42	3.36E-41
TMCC1-AS1	-2.166385	0.6410992	26.19244	3.09E-07	6.80E-07
RAD21-AS1	-2.1613934	-0.3488021	20.28763	6.66E-06	1.34E-05
DLG1-AS1	-2.155218	0.27516476	31.7253	1.78E-08	4.19E-08
SLC7A11-AS1	-2.109168	0.20467094	21.59551	3.37E-06	6.89E-06
GATA2-AS1	-2.0658941	3.6242737	157.4672	4.05E-36	2.42E-35
ATP6V0E2-AS1	-2.0488416	2.38661823	87.83574	7.11E-21	2.87E-20
TTN-AS1	-2.0139936	2.20408455	83.91217	5.17E-20	2.02E-19
YTHDF3-AS1	2.04450591	0.435614	22.1437	2.53E-06	5.24E-06
PROSER2-AS1	2.05225572	-0.5831807	17.34125	3.12E-05	5.94E-05
USP2-AS1	2.21773544	1.69010765	69.58068	7.34E-17	2.57E-16
HOXB-AS2	2.2689812	2.06783222	71.45503	2.84E-17	1.01E-16
UCA1	2.38618236	0.17643564	28.81397	7.97E-08	1.81E-07
FLNB-AS1	2.45010855	2.24877329	112.2863	3.09E-26	1.46E-25
LINC01270	2.46134723	0.64972576	42.44412	7.27E-11	1.95E-10
ACTA2-AS1	2.58754805	-0.5095267	23.36127	1.34E-06	2.83E-06
PRKCQ-AS1	2.60479978	0.77125349	53.93525	2.07E-13	6.21E-13

TMEM105	2.71565074	0.08890389	43.19678	4.95E-11	1.34E-10
LINC00242	2.77206837	-0.1552829	32.19969	1.39E-08	3.30E-08
PRKAG2-AS1	2.86094256	0.28065509	37.05177	1.15E-09	2.89E-09
C22orf24	2.96950452	0.46974239	48.62469	3.10E-12	8.80E-12
FOXD2-AS1	3.03085521	0.7879828	54.94699	1.24E-13	3.75E-13
HOXB-AS3	3.05446853	4.12666276	369.4462	2.47E-82	2.83E-81
SPATA3-AS1	3.20469336	0.59862776	53.28281	2.89E-13	8.59E-13
PRR34-AS1	3.23064761	0.40108666	56.48829	5.65E-14	1.74E-13
MCF2L-AS1	3.44081553	-0.298751	44.77169	2.21E-11	6.08E-11
AC253572.1	3.48122223	-0.6596533	35.57113	2.46E-09	6.08E-09
EML2-AS1	3.62068917	-0.3140591	35.14455	3.06E-09	7.55E-09
LINC01137	3.62393559	0.18026269	67.92038	1.70E-16	5.86E-16
LINC00910	3.68802354	-0.4924737	42.48805	7.11E-11	1.91E-10
CRNDE	3.75416768	1.91374332	170.5728	5.55E-39	3.51E-38
AC093642.5	3.80625366	0.01661847	59.40207	1.29E-14	4.07E-14
FLJ20021	3.81601282	0.33115083	53.34696	2.80E-13	8.32E-13
LINC01315	3.86483799	0.74299536	82.43205	1.09E-19	4.23E-19
LINC01140	3.88312893	1.32052012	122.5908	1.71E-28	8.62E-28
AGAP2-AS1	3.89773365	0.99665128	97.91908	4.36E-23	1.90E-22
LINC00659	3.90369882	0.91090347	67.46692	2.14E-16	7.33E-16
HAGLR	3.9389709	2.03019082	174.9438	6.16E-40	3.97E-39
LINC00319	4.29068634	-0.2528894	59.44659	1.26E-14	3.98E-14
MIRLET7BHG	4.44642883	2.93042534	330.785	6.48E-74	6.71E-73
TOLLIP-AS1	4.57732295	-0.3431712	54.85372	1.30E-13	3.93E-13
ITGB2-AS1	4.76282327	-0.5844758	25.58193	4.24E-07	9.24E-07
ELFN1-AS1	4.84902965	2.21792321	263.891	2.43E-59	2.09E-58
FAM225B	4.9623989	1.05300094	138.29	6.30E-32	3.43E-31
LINC00628	5.06226732	-0.3743036	57.41631	3.53E-14	1.10E-13
FAM225A	5.07307886	0.93934321	138.2278	6.50E-32	3.54E-31
HOXB-AS4	5.10097483	-0.3431124	64.11386	1.17E-15	3.89E-15
KCNK4-TEX40	5.19942485	1.72139293	16.44877	5.00E-05	9.37E-05
AC007128.1	5.22548187	-0.2355214	64.55064	9.41E-16	3.13E-15
LINC00649	5.2265057	2.01227105	254.8822	2.24E-57	1.88E-56
PRRT3-AS1	5.23141675	0.58628973	91.7489	9.84E-22	4.11E-21
MIR194-2HG	5.25399084	-0.207162	67.6979	1.91E-16	6.54E-16
MIR34AHG	5.3962623	2.05685512	251.9808	9.61E-57	7.98E-56
OR2A1-AS1	6.06135342	0.52133238	121.8082	2.54E-28	1.27E-27
LINC00511	6.12417297	2.32482086	328.5316	2.01E-73	2.06E-72
BBOX1-AS1	6.2268846	-0.0555484	61.1514	5.29E-15	1.70E-14

AC108142.1	6.29432794	0.72904656	133.1066	8.57E-31	4.56E-30
SATB2-AS1	6.69566132	0.36587357	112.8584	2.32E-26	1.10E-25
LINC00960	6.93377841	1.83255871	253.7922	3.87E-57	3.24E-56
LINC01094	7.07580309	-0.9371012	39.76967	2.86E-10	7.41E-10
HAR1A	7.21113255	0.84960679	118.1564	1.60E-27	7.86E-27
LINC01152	7.25790479	0.88436217	120.2196	5.66E-28	2.80E-27
ALDH1L1-AS2	7.44809395	-0.6207319	55.14149	1.12E-13	3.41E-13
LINC00494	7.66326089	-0.4402804	63.13612	1.93E-15	6.34E-15
LINC00668	7.79309282	-0.3149664	41.71942	1.05E-10	2.79E-10
LINC00543	7.80598052	-0.3126289	73.5605	9.76E-18	3.53E-17
GATA6-AS1	7.82657639	-0.2964579	73.37792	1.07E-17	3.86E-17
LINC01133	7.84420032	-0.2811716	76.21798	2.54E-18	9.38E-18
HOXA-AS2	7.84487851	-0.2809964	74.36832	6.48E-18	2.36E-17
C1QTNF1-AS1	7.90951797	-0.2224511	78.12512	9.67E-19	3.63E-18
SOX9-AS1	7.92686777	-0.2077262	78.39124	8.45E-19	3.18E-18
AC005538.3	8.02405629	-0.1232388	70.74665	4.06E-17	1.43E-16
LINC01123	8.04045195	1.64239447	260.5908	1.28E-58	1.09E-57
ZNF503-AS2	8.04847796	-0.097915	88.19107	5.94E-21	2.41E-20
HOXA11-AS	8.37236804	0.1924162	96.48643	8.99E-23	3.87E-22
LINC00920	8.37264898	0.19239038	84.03232	4.87E-20	1.91E-19
SH3PXD2A-AS1	8.43791355	0.25505399	97.97487	4.24E-23	1.85E-22
LINC00526	8.47780722	0.29448084	110.4402	7.85E-26	3.68E-25
PAX8-AS1	8.62575823	2.98746917	443.4539	1.92E-98	2.60E-97
SSTR5-AS1	8.65802149	0.45287809	64.05255	1.21E-15	4.01E-15
ADIRF-AS1	8.96216055	2.54515649	368.2407	4.52E-82	5.15E-81
AC005256.1	9.11885524	0.89371764	158.9393	1.93E-36	1.16E-35
LINC01508	9.12638364	0.90439885	128.8661	7.26E-30	3.76E-29
HOXA10-AS	9.172448	0.94224665	148.74	3.27E-34	1.88E-33
CASC8	9.20364479	0.97330441	162.4095	3.37E-37	2.06E-36
SNHG18	9.31838873	1.08327517	165.1482	8.49E-38	5.24E-37
TRPM2-AS	9.35316595	1.1174025	176.0231	3.58E-40	2.32E-39
LINC01559	9.44103	1.19885094	163.803	1.67E-37	1.03E-36
HEIH	9.51445186	1.27145624	199.3376	2.91E-45	2.04E-44
AGAP11	9.66754781	1.42390784	110.9611	6.03E-26	2.84E-25
LINC00941	9.74431482	1.49305731	213.9151	1.92E-48	1.41E-47
HOTAIRM1	9.82049412	1.56694732	221.6196	4.01E-50	3.02E-49
AP000439.3	10.1697068	1.90907242	277.8517	2.21E-62	1.98E-61
CASC19	10.1790361	1.91608993	244.1971	4.78E-55	3.88E-54
LINC01106	10.1804225	1.91937466	295.7766	2.74E-66	2.59E-65

LINC00667	10.2200554	1.95715874	246.504	1.50E-55	1.23E-54
LINC00460	10.250303	1.98763791	318.621	2.89E-71	2.91E-70
MNX1-AS1	10.3626007	2.09757336	321.6486	6.34E-72	6.40E-71
AFAP1-AS1	11.6809185	7.45745497	2235.238	0	0

Supplementary Table S2. Clinical features of the age, stages, and grades in different groups.

**Group (samples)**	**All (n=374)**	**Training (n=188)**	**Testing (n=186)**	
Clinical features	n (%)	n (%)	n (%)	p-value
Age				0.8782
<=65	257 (68.72%)	128 (68.09%)	129 (69.35%)	
>65	117 (31.28%)	60 (31.91%)	57 (30.65%)	
Stage				0.2956
Stage I	1 (0.27%)	1 (0.53%)	0 (0%)	
Stage II	22 (5.88%)	11 (5.85%)	11 (5.91%)	
Stage III	291 (77.81%)	139 (73.94%)	152 (81.72%)	
Stage IV	57 (15.24%)	34 (18.09%)	23 (12.37%)	
unknown	3 (0.80%)	3 (1.6%)	0 (0%)	
Grade				0.0134*
Grade I	1 (0.72%)	1 (0.53%)	0 (0%)	
Grade II	42 (11.23%)	30 (15.96%)	12 (6.45%)	
Grade III	320 (85.56%)	151 (80.32)	169 (90.86)	
Grade IV	1 (0.27%)	1 (0.53%)	0 (0%)	
unknown	10 (2.67%)	5 (2.66%)	5 (2.69%)	

Note: Three groups were the entire (All), control (Train), and validation (Test) groups. *, The difference was analyzed by the Wilcoxon rank-sum test and was statistically significant when a p-value was less than 0.05.

Supplementary Table S3. Prediction of prognostic signatures in ovarian cancer.

**Predictive Models**	**Details**	**PMID #**
Clinical features	Advanced age	30157707
	Subclassify IV FIGO stage	29324537<br>36765667<br>37564940
	lymph node metastases	30976643
Serological parameters	CA-125	33403051
	HE4	16607372
	Ova1	20664381
	VEGF	15154640
	Kallikreins	21412167

	Osteopontin	16764622
	Mesothelin	16428485
	M-CSF	9815777
	Bikunin	15735122
	EphA2	18443431
	Transthyretin	29609043
	Transferrin receptor 1	28319068
	B7-H4	27073557
	Prostatin	11584061
	EGF receptor	20066160
	ctDNA	37762691
	Gene signature	35769257
Imaging examination	Mathematical descriptors	30770825
	CT features	28641043
Combined parameters	CA125, HE4, MSL, and surgical outcome	37816681
	Clinical, serological, and radiomic biomarkers	37875466

PMID, PubMed identifier (https://pubmed.ncbi.nlm.nih.gov/)

Supplementary Table S4. Current lncRNA biomarkers in ovarian cancer.

lncRNA Biomarkers	lncRNAs	PMID #
Pyrptosis-related lncRNAs	MYCNOS, AL161772.1, USP30-AS1, ZNF32-AS2, AC068733.3, AC012236.1, AC015802.5, KIAA1671-AS1, AC013403.2, MIR223HG, KRT7-AS, PTPRD-AS1 and LINC01094	37859811
Immune-related lncRNAs	MYCNOS、CTD-2595P9.4、RP11-1094M14.8和RP5-991G20.1	37674251
Fatty acid metabolism-related lncRNAs	AC080013.1, GS1-124K5.4, AC099518.1, AC011595.1, LINC00861, AC027279.1, AC145343.1, AL021707.1	37673886
Mitophagy-related lncRNAs	AC007637.1, AC020637.1, AC114741.1, AL513550.1, and LINC00174	37633972
Cuproptosis-related lncRNAs	LINC00189, LINC00861, ZFHX4-AS1, RPS6KA2-IT1, LINC00582, C9orf106, DEPDC1-AS1, LINC01556, LEMD1-AS1, and TYMSOS	37528285
EMT-related lncRNAs	RP11-295G20.2, RP11-469H8.6, AL022344.7, RP11-438B23.2, and LINC01224	37390286
Methylation and immune-related lncRNAs	LINC00189, LINC00664, LEMD1-AS1, C9orf106, GAS5, IFNG-AS1, TYMSOS, OCIAD1-AS1, RPS6KA2-IT1, PCOLCE-AS1	37257331
M5C-related lncRNAs	AC005562.1, AC036103.1, AC074029.3, AL139815.1, AL590652.1, CACNA1C-AS1, LNC-LBCS, MIR600HG, WAC-AS1	37183262
Oxeiptosis-related lncRNAs	HOXB-AS3, AC009097.2, AL359220.1, AC100861.1, AC245884.9	37211398
Ferroptosis-related lncRNAs	AC007796.1, TLR8-AS1, RP11-713M15.2, CTB-171A8.1, LBX2-AS1, CTD-2130F23.2, RP11-88G17,6, RP11-388M20.1, RP11-678G14.3, RP4-650F12.2, RP11-701H24.7, RP11-1018N14.5, LINC01281, RP11-301G19.1, CTD-2330K9.3, AP000344.3, CTD-2506J14.1, and AC078842.3	36830675
Immune-related lncRNAs	AL391832.3, LINC00892, LINC02207, LINC02416, PSMB8.AS1, AC078788.1 and AC104971.3	36739404
Immune-related lncRNAs	UBXN10-AS1, TOPORS-AS1, HIPK1-AS1, CELSR3-AS1 and CECR5-AS1	36313727
Histone acetylation-related	LEMD1−AS1, AC138904.1, AC010422.2, AC021016.1, LINC02321, AC008752.1,	36313424

lncRNAs	AL590652.1, AL157871.2, AC092171.2, AC060766.6, AC093734.1, AC011445.1, AC040169.1, UBE2Q1−AS1	
M6A-related	WAC-AS1, LINC00997, DNM3OS and FOXN3-AS1	36011053
Necroptosis-related lncRNAs	AP003392.3, AL928654.1, AL133371.2, AC007991.4, AC011445.1, LINC00996	35965557
Ferroptosis-related lncRNAs	RP11-443B7.3, RP5-1028K7.2, TRAM2-AS1, AC073283.4, RP11-486G15.2, RP11-95H3.1, RP11-958F21.1, and AC006129.1	35756659
Immune-related lncRNAs	KRT7-AS, USP30-AS1, AC011445.1, AP005205.2, DNM3OS and AC027348.1	35031063
Glycolysis-related lncRNAs	**AC133644.2, CTD-2396E7.11, CTD-3065 J16.9, LINC00240, TMEM254-AS1**	34560889
Immune-related	**C007406.4, AC008750.1, AL022341.2, AL133351.1, FAM74A7, LINC02229, and HOXB-AS2**	34367253

PMID, PubMed identifier (https://pubmed.ncbi.nlm.nih.gov/)

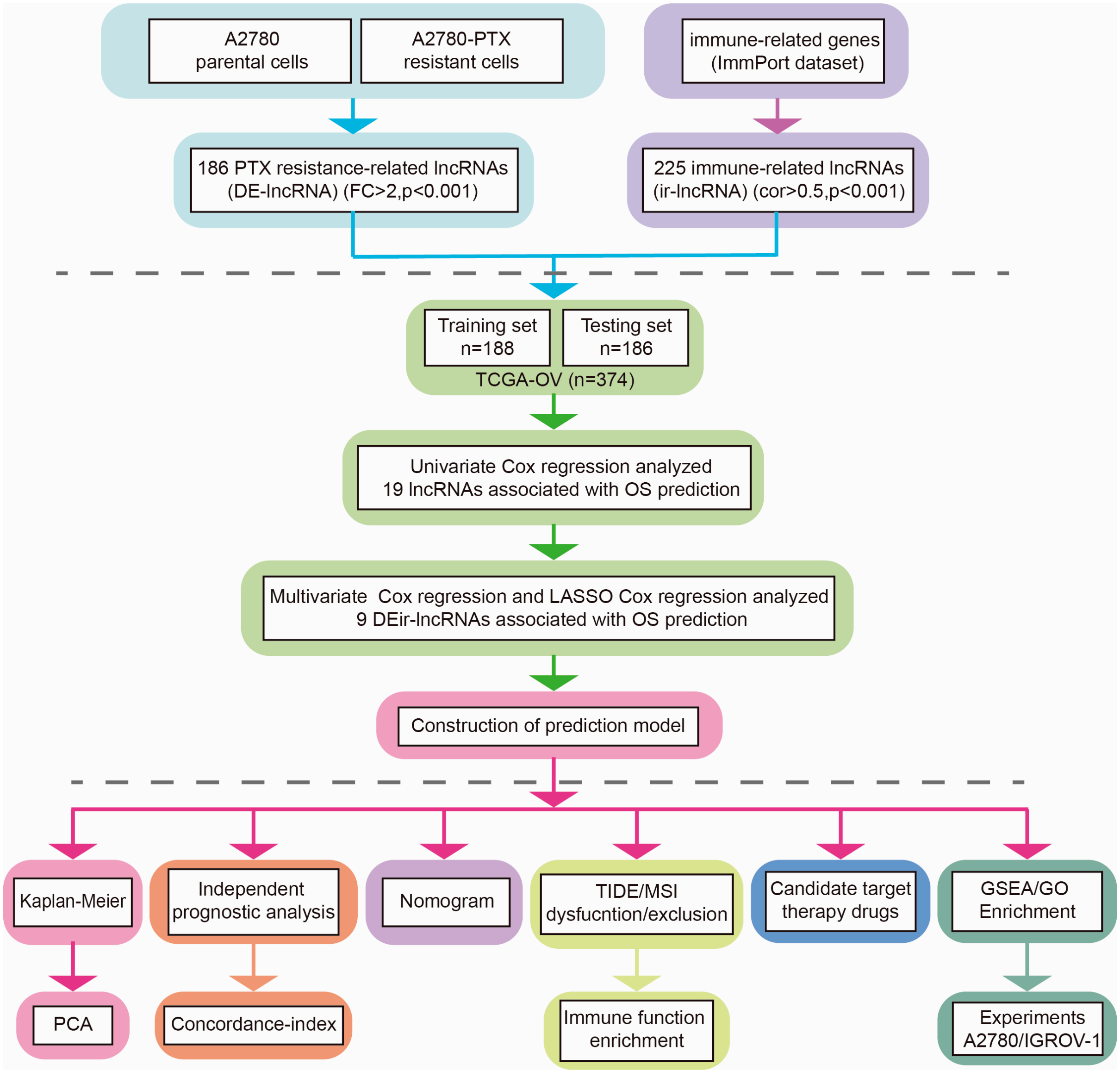

Supplementary Figure S1. Flowchart of the study.

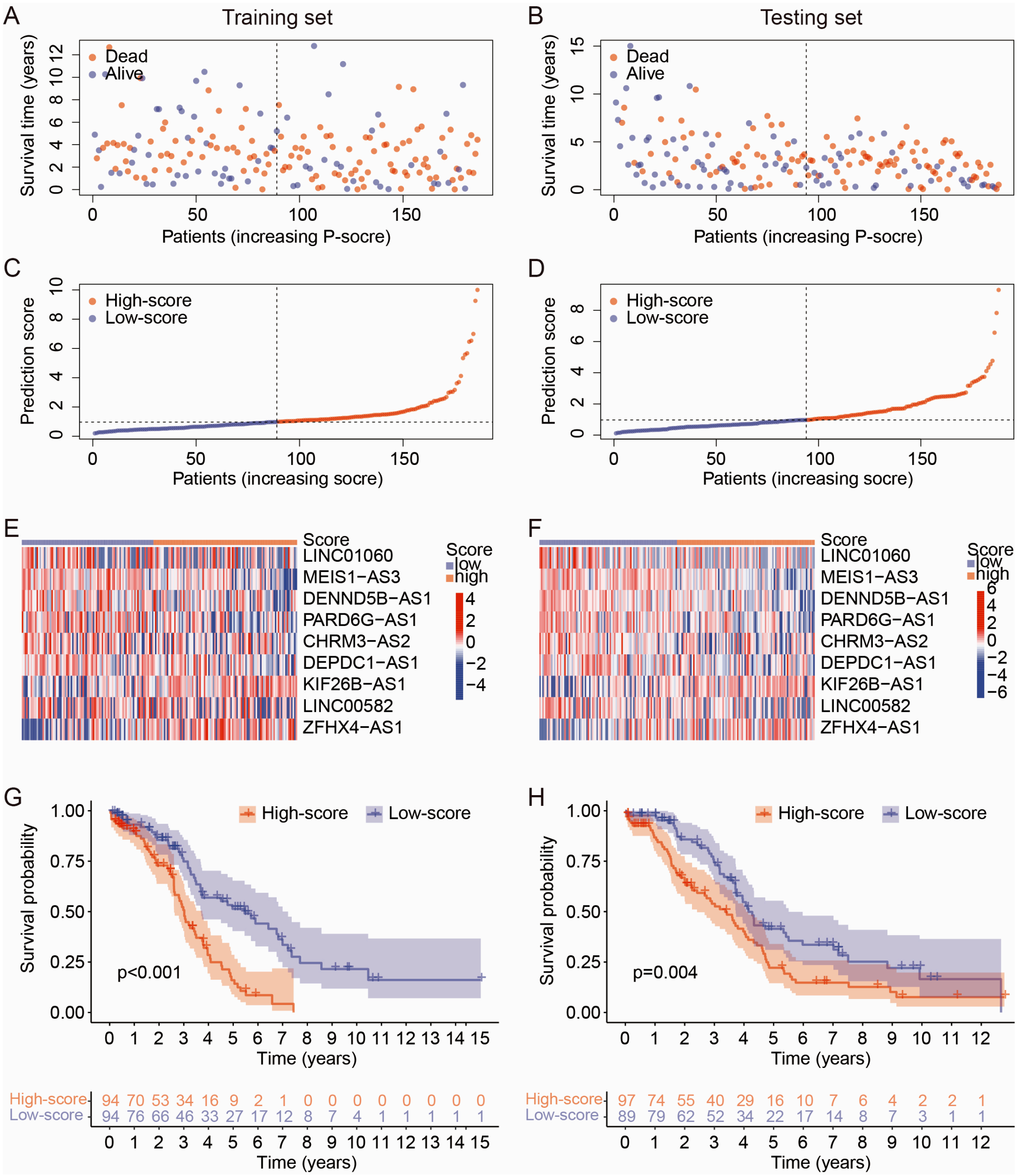

Supplementary Figure S2. Validation of the prediction model in Testing and Training subsets. (A, B) Distribution of survival time between high- and low-score groups in the testing set and training set. (C, D) Distribution of prediction scores between high- and low-score groups in the testing set and training set. (E, F) Heatmap showing expression levels of prediction model-related lncRNAs in the testing set and training set. (G, H). Kaplan-Meier plot showing survival probability between high- and low-score groups in the testing set and training set. A p-value < 0.05 was considered statistically significant.

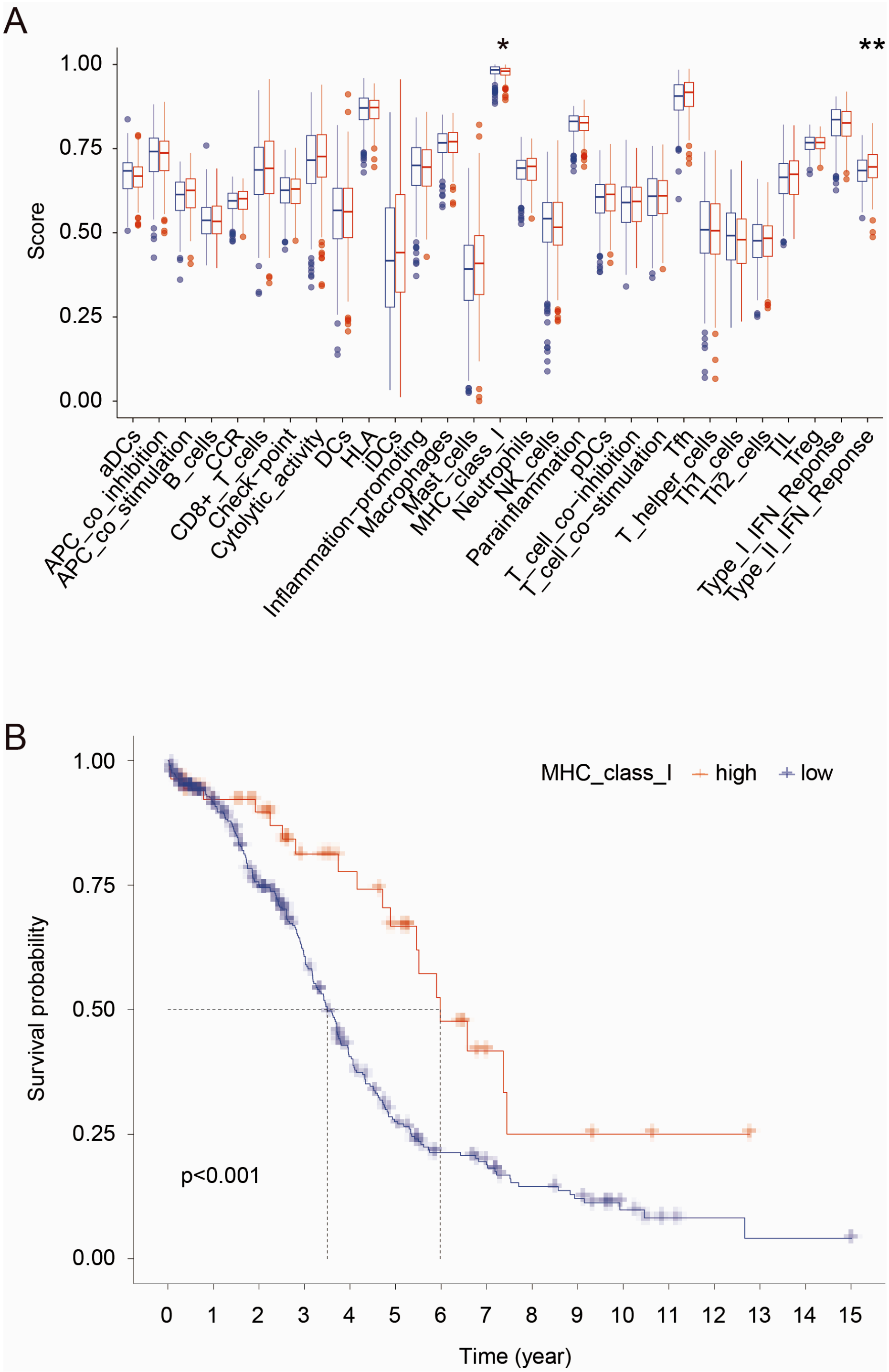

Supplementary Figure S3. Analysis of immune function and correlation. (A) Immune function value analysis between high- and low-score groups. (B) Kaplan-Meier plot showing the correlation of MHC_class_I with the overall survival of patients. A p-value < 0.01 was considered statistically significant. MHC, major histocompatibility complex.

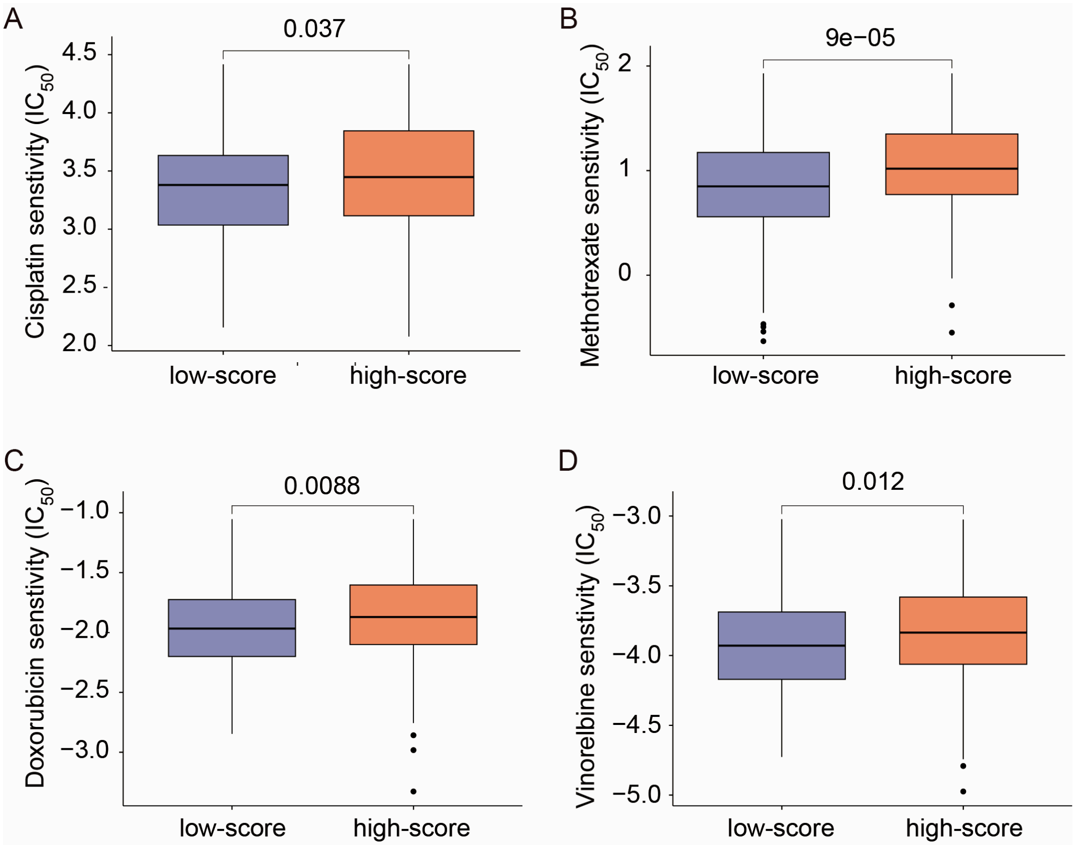

Supplementary Figure S4. Sensitivity of chemotherapy drugs. Box plot showing the Half-maximal inhibitory concentration (IC_{50}) of Cisplatin (A), Methotrexate (B), Doxorubicin (C), and Vinorelbine (D) between high- and low-score groups. A p-value < 0.05 was considered statistically significant.
